# Extraction and Analysis by HPLC-DAD of Carotenoids in Human Faeces from Spanish Adults

**DOI:** 10.3390/antiox9060484

**Published:** 2020-06-03

**Authors:** Elena Rodríguez-Rodríguez, Beatriz Beltrán-de-Miguel, Kerly X. Samaniego-Aguilar, Milagros Sánchez-Prieto, Rocío Estévez-Santiago, Begoña Olmedilla-Alonso

**Affiliations:** 1 Facultad de Farmacia, Universidad Complutense de Madrid, 28040 Madrid, Spain; elena-rd79@farm.ucm.es (E.R.-R.); beabel@farm.ucm.es (B.B.-d.-M.); 2 Institute of Food Science, Technology and Nutrition (ICTAN-CSIC), 28040 Madrid, Spain; kerlysam@ucm.es (K.X.S.-A.); msprieto@ictan.csic.es (M.S.-P.); rocio.estevez@ufv.es (R.E.-S.); 3 Facultad de Ciencias Experimentales, Universidad Francisco de Vitoria, 28223 Pozuelo de Alarcón (Madrid), Spain

**Keywords:** carotenes, xanthophylls, diet, stool, bioavailability, middle-age people

## Abstract

Carotenoids are bioactive compounds with widely accepted health benefits. Their quantification in human faeces can be a useful non-invasive approach to assess their bioavailability. Identification and quantification of major dietary carotenoids in human faeces was the aim of the present study. Faeces and dietary intake were obtained from 101 healthy adults (45–65 years). Carotenoid concentrations were determined by HPLC in faeces and by 3-day food records in dietary intake. Carotenoids quantified in faeces (μg/g dry weight, median) were: β-carotene (39.5), lycopene (20), lutein (17.5), phytoene (11.4), zeaxanthin (6.3), β-cryptoxanthin (4.5), phytofluene (2.9). α-carotene (5.3) and violaxanthin were found 75.5% and 7.1% of the faeces. The carotenoids found in the highest concentrations corresponded to the ones consumed in the greatest amounts (μg/d): lycopene (13,146), phytoene (2697), β-carotene (1812), lutein+zeaxanthin (1148). Carotenoid concentration in faeces and in dietary intake showed correlation for the total non-provitamin A carotenoids (r = 0.302; *p* = 0.003), phytoene (r = 0.339; *p* = 0.001), phytofluene (r = 0.279; *p* = 0.005), lycopene (0.223; *p* = 0.027), lutein+zeaxanthin (r = 0.291; *p* = 0.04) and β-cryptoxanthin (r = 0.323; *p* = 0.001). A high proportion of dietary carotenoids, especially those with provitamin A activity and some of their isomers, reach the large intestine, suggesting a low bioavailability of their intact forms.

## 1. Introduction

Carotenoids have been widely studied owing to their beneficial properties on health. In addition to the provitamin A activity that some exhibit, there are also others related to their anti-oxidant, anti-inflammatory, photo-protective and immunological capacity [1,2]. The principal sources of carotenoids are fruits and vegetables but their bio-accessibility from these sources is low. Some studies have found that approximately 70% of carotenoids may actually remain in the final digesta [3,4,5]. This means that significant concentrations could reach the colon. However, it is not known whether carotenoids can be further taken up or whether a significant proportion is fermented to unknown metabolites. This is important as studies indicate that polar metabolites and/or derivatives appear to be bioactive and interact with transcription factors such as NF-κB and Nrf2 and nuclear receptors such as RXR/RAR [2]. While it is known that a fraction of carotenoids taken up by the enterocytes can be cleaved by beta-carotene oxygenases (BCO1/2) into apo-carotenoids, there is scant information about further metabolism, biodistribution or excretion [6]. Faeces provide a valuable matrix to study changes during in vivo digestion and the bioavailability of dietary components [7]. However, few studies on the identification and quantification of carotenoids have been conducted using human faeces [4,7,8,9].

The validity of analysing carotenoid content in microsamples of human faeces as a non-invasive approach in nutritional studies was confirmed in an intervention study with a β-carotene enriched beverage given to 40 post-menopausal women and 11 pre-term infants [4]. Recently, a suitable method was set up and validated to determine dietary isoprenoids in the faeces of five-month-old babies fed with purees made of different vegetables [7]. In addition, faecal samples have already been used to assess carotenoids bioavailability in a few studies to date, one focusing on β-carotene in 17 Indonesian children [8] and another on β-cryptoxanthin and major dietary carotenoids in an intervention study involving 40 post-menopausal women [9]. However, data regarding carotenoid content in human faeces samples to date comes from studies with a small number of participants and the relationship between the dietary intake of carotenoids and their presence in faecal matter has yet to be studied in depth. In this connection, our study stands out for the high number of well characterized Spanish subjects between age 45 and 65.

The aim of this work was to evaluate the carotenoid profile in the faeces of apparently healthy adults and compare that with their dietary intake, as this could be a non-invasive approach by which to assess carotenoid bioavailability.

## 2. Materials and Methods

### 2.1. Samples

Samples were obtained from an analytical observational study assessing lutein and zeaxanthin dietary intake and short- and long-term status markers (serum concentrations and macular pigment optical density) to assess their predictive value for macular pigment optical density and visual function in apparently healthy subjects [10].

A total of 101 subjects (77 women and 24 men) were recruited. Inclusion criteria were: age range 45–65 years, cholesterolemia within normal range (upper limit 240 mg/dL), body mass index ≥ 20 and ≤30 kg/m^2^, varied diet (no avoidance of any food groups) and, among exclusion criteria: consumption of food supplements, use of drugs or phytosterol-enriched beverages/foods to control cholesterolemia, regular consumption of n-3 fatty acid-enriched food products and chronic diseases that can affect carotenoid or lipid metabolism (i.e., diabetes, cardiovascular disease).

Faeces samples were collected and kept in the fridge until delivery to the lab the same day of collection or were frozen at −20 °C until delivery (maximum 1 month). Once received in the laboratory, they were kept at −80 °C, for a maximum of 3 months, under N_2_ atmosphere until lyophilisation. Samples were then put in the freeze-drier for 48 h at −70 °C. Once lyophilised, they were returned to the freezer at −80 °C until extraction and analysis.

Recent dietary intake was evaluated using 3-day food records involving 24 h recalls, one of which coincided with a weekend or holiday, carried out within a period of 7 to 10 days.

The study was approved by the Ethical Committee of Research with Drugs of the Hospital Universitario Puerta de Hierro Majadahonda of Madrid, Spain (acta no. 03.17, dated 13 February 2017) and by the CSIC’s Ethics Committee, Bioethic Subcommittee (dated 21 February 2017). All subjects signed a written informed consent for joining the study.

### 2.2. Chemicals and Standards

Lutein (xanthophyll from marigold), zeaxanthin, α- and β-carotene, β-cryptoxanthin, lycopene and phytoene, tocopheryl acetate, trimethylamine, celite, potassium hydroxide (KOH), phosphate buffered saline (PBS) and sodium chloride (NaCl) were obtained from Sigma Aldrich (Madrid, Spain). Sodium sulphate anhydrous and pyrogallic acid were supplied by Panreac (Barcelona, Spain). Hexane (Hex), methyl tert-butyl ether (MTBE), methanol (MeOH), ethanol, dichloromethane (DCl), petroleum ether (PE) and diethyl ether (DE), were obtained from Análisis Vínicos (Spain). MeOH and MTBE were HPLC grade.

### 2.3. Carotenoid Extraction

#### 2.3.1. Rehydration of Faeces Samples

A preliminary assay was conducted to select the lyophilised sample rehydration procedure. One faeces sample, in triplicate (0.2 g) was hydrated in three different ways: (1) with 10 mL distilled H_2_O + 2 mL ethanol; (2) 10 mL NaCl 10% + 2 mL ethanol; and (3) 10 mL phosphate buffered saline (PBS) 1% + 2 mL ethanol. After 20 min of rehydration, the carotenoids were extracted with DE:PE (50:50) as the extraction solvent as described further on.

#### 2.3.2. Selection of the Extraction Solvent

Before definitive extraction, we analysed in triplicate the capacity of four different solvents to extract the carotenoid compounds from the faeces. Solvent A: DE:PE (1:1); solvent B: MeOH:MTBE:DE (1.2:1.2:1); solvent C: DE; and solvent D: Hex:DCl (5:1).

To extract the carotenoids, we homogenized approximately 0.3 g of the lyophilised faeces sample with 10.0 mL of cool PBS 1x solution (pH = 7.4) and 2 mL of ethanol to hydrate the faeces and magnetically stirred the mixture for 20 min. Then, 20 mL of pure acetone and 750 μL of internal standard (tocopherol acetate 0.32 mg/mL) were added. This was stirred for 4 min on a heat-free plate and then left to rest for one minute. Supernatant liquid was then removed to a 100 mL beaker by decantation and faeces samples were re-extracted with 10 mL of pure acetone and stirred for 4 min. 10 mL of extraction solvent (solvent A) were then added, stirred for 2 min and then centrifuged at 3500 rpm for 3 min. After recovering the coloured fraction, the residue was extracted again with another aliquot of 10.0 mL extraction solvent. This procedure was repeated until the colour disappeared (usually 2 extractions but up to 5 in some cases) and the supernatants were combined in a beaker and anhydrous sodium sulphate was added little by little while swirling the flask to absorb all the aqueous content. The organic coloured fractions were evaporated to dryness in a rotary evaporator at a temperature below 35 °C and dissolved in 25 mL of MeOH:MTBE (50:50), were filtered under membrane filtration (0.45 μm pore size) and transferred to vials which were kept at −20 °C under nitrogen atmosphere until analysis.

### 2.4. Saponification

A previously described fast saponification protocol [11] was used to release hydrolysed xanthophyll fatty acid esters. Briefly, 400 μL of the extracted carotenoids from the faeces were added to a test tube. The same quantity (400 μL) of pyrogallic acid in ethanol (0.1 M) and KOH in MeOH (30%) was then added and placed in an ultrasonic bath in darkness for 7 min. Then, 800 μL of distilled water and 1600 μL of extraction solvent were added. After vortex for 1 min and centrifuge for 3 min at 3500 rpm, the organic phase (supernatant) was transferred to another test tube. This process (from the point at which distilled water and extraction solvent were added) was repeated 2 more times. The organic phase collected was dried under nitrogen and dissolved in 150 μL of MeOH:MTBE (50:50) in preparation for HPLC analysis.

### 2.5. HPLC-DAD Carotenoid Analysis

Carotenoid concentrations were determined by high performance liquid chromatography (HPLC) using a system consisting of a model 600 pump, a Rheodyne injector and a 2998 photodiode array (PDA) detector (Waters, Milford, MA, USA) and a C30 YMC column (5 μm, 250 × 4.6 mm i.d.) (Waters, Wilmington, MA, USA) with a guard column (Aquapore ODS type RP-18) at room temperature (25 °C). Mobile phase was formed by MeOH with 0.1% trimethylamine (solvent A) and MTBE (solvent B) in a linear gradient. At baseline, 25, 55 and 60 min the ratios of the solvents were 70:30, 35:65 and 95:5. The detection was performed at a wavelength of 450 nm for carotenoids, 285 nm for both phytoene and tocopheryl acetate (internal standard) and 270 nm for phytofluene. Chromatograms were processed using Empower 2 software (Waters, Milford, MA, USA).

Identification of trans-carotenoids (all-*E* carotenoids) was based on the retention time (RT) of available standards and comparison with absorption spectra reported in the literature. β-carotene and phytofluene isomers were identified by comparing the RT and spectra obtained with those described in the literature. Standard carotenoid concentrations were calculated on the basis of published extinction coefficients (E 1% 1 cm) in ethanol or petroleum ether [12]. Values used and the wavelength maxima were: lutein, 2550 at 445 nm; zeaxanthin, 2540 at 450 nm; β-cryptoxanthin, 2386 at 452 nm; lycopene 3450 at 472 nm; α-carotene, 2800 at 444 nm; β-carotene, 2592 at 453; neoxanthin 2320 at 437 nm; violaxanthin 2443 at 441 nm.

Carotenoids were quantified using calibration curves for lutein, zeaxanthin, β-cryptoxanthin, α-carotene, β-carotene, lycopene and violaxanthin, at four concentration levels. No standard was available for phytofluene so it was tentatively identified by comparing the RT and spectra obtained with those described in the literature and quantified against a phytoene standard.

The concentrations of the carotenoids in the most diluted curve were: 0.19–1.5 ng/μL for lutein (R^2^ = 0.994), 0.15–1.20 ng/μL for zeaxanthin (R^2^ = 0.996), 0.23–2.10 ng/μL for β-cryptoxanthin (R^2^ = 0.995), 0.11–0.90 ng/μL for α-carotene (R^2^ = 0.996), 0.21–2.10 ng/μL for β-carotene (R^2^ = 0.972) and 0.23–1.80 ng/μL for lycopene (R^2^ = 0.987). Two additional curves were used for higher concentrations. The concentrations of the carotenoids in the most concentrated curve were: 2.62–13.12 ng/μL for lutein (R^2^ = 0.992), 7.26–36.6 ng/μL for zeaxanthin (R^2^ = 0.990), 2.3–11.50 ng/μL for β-cryptoxanthin (R^2^ = 0.980), 1.59-7.94 ng/μL for α-carotene (R^2^ = 0.996), 7.89–19.72 ng/μL for β-carotene (R^2^ = 0.999) and 2.59–12.93 ng/μL for lycopene (R^2^ = 0.999). The concentrations range for violaxanthin in the curve was: 1.15–11.40 ng/μL (R^2^ = 0.989). The equations of these calibration curves are shown in Table 1.

### 2.6. Calculation of Carotenoid Concentrations in Faeces

The amount of each carotene extracted (α-carotene, β-carotene, lycopene, phytoene and phytofluene) was calculated on a dry-weight basis using the following Equation (1):

(1) $$Carotenoid\;\left(\frac{\mu g}{g}\right)=\frac{\mathrm{ng}\;\mathrm{carotenoid}\;\times \;V_{r}\left(\mu L\right)}{V_{injection}\left(\mu L\right)\;\times \;W_{t}\left(g\right)\;\times \;1000}$$

where ng carotenoid is the quantity of each carotenoid calculated from the standard calibration curve in ng, *V_r_* the reconstitution volume (2000 μL), *V_injection_* is 50 μL and *W_t_* the weight of the fresh sample (an appropriate amount: 0.25–0.35 g).

The amount of each xanthophyll extracted after saponification (lutein, zeaxanthin, β-criptoxanthin and violaxanthin) was calculated on a dry weight basis using the following Equation (2):

(2) $$Xantophill\;\left(\frac{\mu g}{g}\right)=\frac{\mathrm{ng}\;\mathrm{xantophyll}\;\times \;V_{r}\left(\mu L\right)\;\times \;V_{rs}\left(\mu L\right)}{V_{injection}\left(\mu L\right)\;\times \;V_{s}\left(\mu L\right)\;\times \;W_{t}\left(g\right)\;\times \;1000}$$

where the ng of carotenoid is the quantity of each carotenoid calculated from the standard calibration curve in ng, *V_r_* is the reconstitution volume (2000 μL), *V_rs_* the reconstitution volume of the saponified sample (150 μL), *V_injection_* is 50 μL, *V_s_* is the volume of sample saponified (400 μL) and *W_t_* is the weight of the fresh sample (0.3 g approx.).

The carotenoid concentration in hydrated faeces (fresh) was calculated bearing in mind the percentage of weight lost during lyophilisation (faeces were weighed before and after lyophilisation).

The concentration of the major carotenoids eliminated in faeces in 24 h was estimated by multiplying carotenoid concentration (μg/g WW) by average bowel movement weight considered to be 128 g/day [13].

### 2.7. Carotenoid Dietary Intake Assessment

Three-day food records involving 24 h recalls, one of which coincided with a weekend or holiday, were used to calculate recent dietary intake. These were recorded within a period of 7 to 10 days. Food consumption (grams/day) was calculated [14] and daily individual carotenoid intake was estimated using a database generated by our group and integrated into a software application [15,16]. Data on phytoene and phytofluene concentration in fruit and vegetables [17] and unpublished data supplied by Dr. A. Meléndez-Martínez were incorporated into our carotenoid database.

### 2.8. Statistical Analysis

The Kolmogorov-Smirnoff test was used to test whether the variables followed normal distribution. None of the variables, except for the ingestion of phytoene and phytofluene, featured a normal distribution and the median and inter-quartile range are presented. The relationship between dietetic and faecal carotenoid data was examined by Pearson’s correlations. A significance level of *p* < 0.05 was used for the analyses. Statistical analyses were performed using the SPSS version 25.0 statistical software package for Windows.

## 3. Results

A total of 98 faeces samples were analysed since 3 of the volunteers either failed to provide a sample or the sample was too small to analyse. Water loss from lyophilisation was 47.0% (36.0–54.5%).

**Figure 1:**
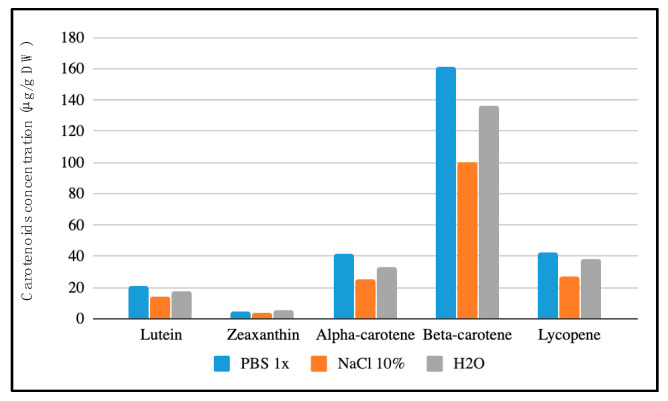
Carotenoids concentration (μg/g dry weight (DW)) in a lyophilised faecal sample extracted with DE:PE after different hydration media.

Figure 1 shows the concentration of the main carotenoids of the diet in dry faeces (μg/g dry weight (DW)) once having hydrated the lyophilised faeces in the 3 different ways described above (distilled H_2_O, NaCl and PBS) and having selected hydration with PBS 1x as it resulted in the best extraction of most of the carotenoids in the study.

Extraction was performed using DE:PE as this was the solvent that extracted the greatest amount of xanthophylls (with no statistical significance) but not of carotenes which was similar for all 4 of the solvents (Table 2).

**Figure 2:**
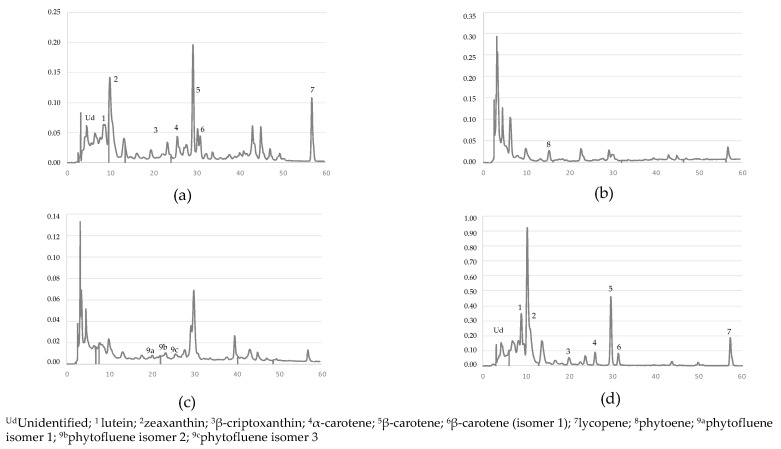
Chromatograms at 450 nm (**a**); 285 nm (**b**) and 370 nm (**c**) of faeces extracted and of the extract saponified at 450 nm (**d**).

A chromatogram of the carotenoid profile extracted from the samples is shown in Figure 2 and the chromatographic and UV-Vis characteristics are shown in Table 3. The carotenoids detected and quantified in faeces were violaxanthin, lutein, zeaxanthin, β-cryptoxanthin, phytoene and phytofluene (3 isomers), α-carotene, all-*E*-β-carotene, β-carotene (isomer 1) and lycopene. At the beginning of the chromatogram, several unidentified compounds eluted before lutein. Peak 2 corresponds to the coelution of zeaxanthin and capsanthin in one of the few faeces samples in which the latter was also present (less than 5%). The zeaxanthin could be quantified because its maximum wavelength (477.3 and 450 nm) is different from that of capsanthin (474.8 nm). As for phytofluene, 3 compounds with similar spectra (330;347;363) appearing around minute 20, 22.5 and 24 were identified, the most frequent and abundant being the one that appeared first (found in 73.5% of the faeces) while the one that appeared last was only found in 12.2% of the faeces. The β-carotene isomer obtained had a spectrum of 413;445.7;472.4 nm and a RT of 33.5 min, always eluting after β-carotene. This isomer was tentatively identified as 9-*Z*-β-carotene based on its absorbance spectrum which showed a hypsochromic shift from the normal β-carotene spectrum and a large *Z* peak at 413 nm, commonly associated with *Z*-carotenoid spectra.

Table 3 shows carotenoid concentrations expressed as median and interquartile range [p25–p75] of concentrations found in the dry faeces samples analysed (μg/g DW). The major carotenoids were found in participants’ faeces were, from highest to lowest: β-carotene, lutein+zeaxanthin, lycopene and phytoene. The other carotenoids were found in almost all faeces in lower concentrations. Violaxanthin was only found in 7.1% of the faeces.

The amount of dietary carotenoids ingested is shown in Table 4. Top on the list was lycopene, followed by phytoene, β-carotene and lutein+zeaxanthin (shown together in this table as individual ingestion data is unavailable). This table also shows dietary carotenoids eliminated in dry faeces (μg/g DW), fresh faeces (μg/g WW) and the estimated amount in faeces after 24 h (μg/24 h).

Assuming that the faeces samples analysed are representative of faeces eliminated after 24 h (which is not the case since faeces samples are not homogeneous), the top carotenoids eliminated on a daily basis would be β-carotene, lutein+zeaxanthin, lycopene and phytoene, and in lower concentrations α-carotene, β-cryptoxanthin and phytofluene. Each carotenoid is eliminated in the same proportion as it is consumed in the diet (Table 4).

There was a significantly positive correlation between dietary carotenoids and the faecal concentrations found (μg/g DW) for β-cryptoxanthin (r = 0.323; *p* = 0.001), lutein+zeaxanthin (r = 0.291; *p* = 0.04), lycopene (0.223; *p* = 0.027), phytoene (r = 0.339; *p* = 0.001) and phytofluene (r = 0.279; *p* = 0.005), but not for α-carotene (r = 0.191; *p* = 0.059), all-*E*-β-carotene (r = 0.094; *p* = 0.358) or for total β-carotene (all-*E* + isomer 1) (r = 0.098; *p* = 0.382). There was a significant correlation between dietary carotenoids and the concentration in fresh faeces and in estimated faecal sample after 24 h for lutein+zeaxanthin (r = 0.227; *p* = 0.025; in both cases), β-cryptoxanthin (r = 0.327; *p* = 0.001; in both cases), phytoene (r = 0.350; *p* = 0.000; in both cases) and phytofluene (r = 0.290; *p* = 0.004; in both cases). In general, when we grouped carotenoids based on whether they exhibit provitamin A activity or not, we found a correlation between the consumption of carotenoids not exhibiting said activity and their concentration in dry faeces (r = 0.302; *p* = 0.003), in fresh faeces and in faeces at 24 h (r = 0.230; *p* = 0.023, in both cases). No correlation was found between the provitamin A carotenoids in dietary intake and in faeces (r = 0.390; *p* = 0.172). Moreover, carotenoids without provitamin A activity were in higher concentrations in dietary intake and in faeces (*p* = 0.000, in both cases).

## 4. Discussion

This study analyses carotenoid content in faeces as a non-invasive approach to carotenoid bioavailability. Faeces samples were lyophilised, stored and later hydrated with three different solvents or mix of solvents. The one affording the best extraction of all the carotenoids was PBS 1x (Figure 1). PBS is an ionic buffer composed of sodium chloride, sodium phosphate, (in some formulations) potassium chloride and potassium phosphate (pH range from 5.8–8.0 at 25 °C), whose osmolarity and ion concentrations correspond to that of human body fluids such as blood [18]. This buffer has been used in other carotenoid studies involving faeces [4,7]. This study focused on the most prevalent carotenoids found in the human diet that are known to have a positive health impact, and on others attracting increasing interest due to their potential health benefits [19]. These carotenoids feature very diverse polarities, carotenes being most soluble in low-polar or apolar solvents (i.e., hexane and petroleum ether), and xanthophylls being more soluble in polar solvents (i.e., ethanol, methanol, acetone) [20,21]. Thus, the first stage of this analysis entailed selecting the most appropriate extraction solvent for the carotenes and xanthophylls present in faeces samples. DE:PE was the solvent that extracted the greatest amount of xanthophylls while the amount of carotenes extracted was similar for all of the solvents tested. This solvent was therefore chosen for carotenoid extraction in the faeces samples.

The carotenoids found in the highest concentrations in faeces (β-carotene, lutein+zeaxanthin, lycopene and phytoene) corresponded to the ones consumed in the greatest amounts by participants. However, high levels of all-*E* and some isomers of non-provitamin A carotenoids were found in faeces compared with their intake. There was a significant correlation between concentrations of β-cryptoxanthin, lutein+zeaxanthin, phytoene and phytofluene in diet and in faeces and the same is true for all the non-provitamin A carotenoids. However, there was no correlation for others such as β-carotene or α-carotene. This could partly be due to their conversion into retinol in the organism and the different losses and isomerizations resulting from food processing, to different degrees of absorption during digestion [22] or to the different degradation and isomerization processes they are subject to during digestion [23,24,25]. Of all these effects, the most important could be the bioconversion of carotenoids to retinol as there is no correlation between provitamin A carotenoids in dietary intake and faeces but there is correlation in the case of non-provitamin A carotenoids.

β-carotene is present mainly as all-*E* in raw foods and in the faeces samples. The lack of significant correlation between its dietary intake and presence in faeces could influence the high variability reported for this carotenoid in in vitro digestion studies (range 30–70%) [23,26]. In this study, large amounts of the isomer tentatively identified as 9-*Z*-β-carotene was found in a high percentage of the faeces samples analysed (79.6%) the all-*E/Z* β-carotene ratio being 3.5. Other studies involving faeces also described the presence of 9-*Z*-β-carotene in lower concentrations than the all-*E* form [4,7]. This isomer elutes immediately after its all-*E* form when similar chromatographic conditions are used [4,27,28]. The importance of the presence of 9-*Z*-β-carotene in faeces should be stressed as this isomer can be metabolized to its respective retinoic acid, 9-*Z* retinoic acid in intestinal mucosa which has been related with gene regulation [29,30] and beneficial health effects such as the inhibition of atherosclerosis by improving cholesterol efflux from macrophages [30]. Furthermore, high β-carotene concentration in faeces and the fact that it does not significantly correlate with intake, could also be due to its presence in the intestine from food consumed the previous day and released during subsequent post-prandial phases [31].

β-crypoxanthin shows the highest correlation between dietary intake and content in faeces. It is also the carotenoid exhibiting the highest correlation between ingestion and serum concentration [32,33]. Its low degree of elimination in faeces is also striking and could be related to high absorption levels and/or a high capacity to convert into retinol. Thus, when studying the apparent bioavailability of the different provitamin A carotenoids, a much higher bioavailability of β-cryptoxanthin-rich foods compared to β-carotene-rich foods (725%) has been described and is most likely due to the presence of one or two hydroxylated group(s) that increase(s) its solubility into the micellar structures [34,35].

Although there was a significant correlation between lycopene intake and faeces concentrations, there is a large difference between those concentrations which could be due to low absorption which has been estimated at 23% [36], to its effect on gut microbiota [37] and even to its degradation and isomerization during digestion. Only one lycopene isomer was found (Figure 2) even though up to five have been described in the faeces of babies fed with tomato puree [7]. More lycopene isomers, up to 10 peaks, have been described in serum and lung samples, formed as a result of thermal stereomutation [38].

Lutein concentration in faeces is higher than that of zeaxanthin (nearly 3 times higher), as is also the case with dietary consumption, i.e., 13 times higher in the Spanish diet [39]. As described by Hernández-Álvarez [4], several unidentified compounds are eluting before the lutein can correspond with different apo-carotenoids. Moreover, metabolic activity in mammals oxidizes the secondary hydroxyl group to a carbonyl group in xanthophylls such that a substantial amount of keto-carotenoids is produced from lutein/zeaxanthin in humans [40]. Some of these keto-carotenoids (such as ε,ε-carotene-3,3′-dione and 3′-hydroxy-ε,ε-caroten-3-one) eluting near the lutein peak have been described in human plasma samples [41].

Phytoene is found in greater amounts in faeces than phytofluene, as is also the case with the dietary amount consumed in this study and in another populational study which estimated the daily per capita intake of phytoene and phytofluene combined accounted for 16% of the total dietary intake of carotenoids [42]. Phytoene is also present in higher concentrations in foods than phytofluene [28].

Three phytofluene isomers were found in the faeces samples and, although they could not be determined directly, we were able to identify them by comparing them with those described by Meléndez-Martinez [38] who identified 6 phytofluene isomers with a wavelength of 370 nm and similar spectra (328;345;363 nm) and with help from the work by Rosso and Mercadante [27] who identified the Z-phytofluene to a RT of 20.4–21 and spectra of 330;347;366 nm and the all-*E*-phytofluene to a RT of 24.4 and the same spectra as *Z*-phytofluene.

Our results show that a high proportion of dietary carotenoids ingested, especially those with provitamin A activity and some of their isomers, generally reach the large intestine, specifically the colon, with the corresponding beneficial health effects. In this connection, several in vitro studies have shown that carotenoids, following colonic fermentation, are not completely recovered but rather are broken down (i.e., fermented) by microbiota [6]. Intestinal microbiota plays a major role in the breakdown, absorption and metabolism of key dietary constituents contributing, for example, to the transformation of compounds to bioactive components [43] as is the case with the metabolization of 9-*Z*-β-carotene to its respective retinoic acid. It has also been shown that some foods, owing to their content in certain nutrients including carotenoids, are able to regulate intestinal microbiota and lower the risk of several chronic diseases [44,45,46].

This study is limited insofar as faeces samples were not homogeneous and therefore a given sample is not representative of a 24 h faeces excretion nor can an adequate correlation be drawn with dietary intake. Taking 24 h faeces samples entails a higher economic compensation than what could be offered in this study. Furthermore, this is a cross-sectional study meaning that causal inferences cannot be made and the possibility of residual or unknown confounding cannot be excluded. Additionally, our study included only one ethnic group and future studies should examine these relationships in other populations. Future research should also include a higher number of individuals from different age brackets and races and 24 h faeces samples. This would allow faeces samples to be used as a complementary marker when assessing bioavailability.

## 5. Conclusions

The high level of the major dietary carotenoids in faeces samples in all-*E*- and some isomers in *Z*-form, especially those with provitamin A activity, was particularly striking, and suggests low bioavailability. However, their presence in the colon could have health benefits as they can metabolize to bioactive compounds and regulate intestinal microbiota thus lowering the risk of several chronic diseases.

## Figures and Tables

**Table 1:** Equations of the calibration curves used in the quantification of carotenoids.

Carotenoids	Equation	
	Calibration Curve (Diluted)	Calibration Curve (Concentrated)
Lutein	y = 8.61 × 10^3^x + 3.08 × 10^3^	y = 1.43 × 10^4^x − 4.22 × 10^5^
Zeaxanthin	y = 1.25 × 10^4^x − 9.04 × 10^2^	y = 1.68 × 10^4^x − 1.36 × 10^6^
β-cryptoxanthin	y = 1.11 × 10^4^x − 2.79 × 10^3^	y = 1.28 × 10^4^x + 4.57 × 10^5^
α-carotene	y = 1.38 × 10^4^x − 3.76 × 10^3^	y = 1.21 × 10^4^x + 2.54 × 10^5^
β-carotene	y = 7.67 × 10^3^x + 3.19 × 10^4^	y = 1.24 × 10^4^x + 9.26 × 10^5^
Lycopene	y = 6.14 × 10^3^x + 1.70 × 10^4^	y = 6.92 × 10^3^x + 1.03 × 10^5^

**Table 2:** Carotenoids concentration (μg/g DW) extracted with different solvents (X ± SE).

Solvent	DE:PE	MeOH:MTBE:DE	DE	Hex:DCl
Carotenes (μg/g DW)	80.10 ± 11.65	81.63 ± 18.54	78.61 ± 19.91	69.12 ± 19.19
Xanthophylls (μg/g DW)	110.79 ± 54.56	34.18 ± 16.65	43.80 ± 27.07	38.11 ± 22.65

X ± SE: mean ± standard error.

**Table 3:** Carotenoids profile and carotenoids concentration (p50 [p25–p75]) in the faecal samples.

Carotenoid	RT (min)	λ max	Concentration (μg/g DW)
*Xanthophylls*			
Violaxanthin	7.2	411.8;434.4;426.7	0 (0-0)
Lutein	10.2	444.5;472.4	17.46 (11.73–30.76)
Zeaxanthin	14.9	450;477.3	6.32 (3.33–13.93)
β-Cryptoxanthin	22.4	450;477.3	4.48 (1.79–7.81)
*Carotenes*			
Phytoene	17.2	284.9	11.37 (4.30–20.00)
Phytofluene isomer 1	20	330;347;363.5	2.04 (0–5.96)
Phytofluene isomer 2	22.5	330;347;363.5	0.81 (0–2.67)
Phytofluene isomer 3	24	330;347;363.5	0 (0-0)
α-Carotene	28.4	444.5;472.4	5.29 (0.53–11.88)
β-Carotene	32.5	450.5;477.3	30.92 (17.25–46.31)
9-*Z-*β-Carotene (isomer 1)	33.5	413;445.7;472.4	9.71 (5.04–12.80)
Lycopene	56	444.5;472.4;501.6	20.02 (9.57–34.55)

RT: retention time; DW: dry weight.

**Table 4:** Dietary and faecal carotenoids concentrations (p50 [p25–p75]).

Carotenoid	Intake (μg/day)	Concentration (μg/g DW)	Concentration (μg/g WW)	Estimated Concentration in Faeces (μg/24 h) *	Estimated Percentage of Loss (%)
Lutein+zeaxanthin	1148 (666–1907)	25.48 (17.46–44.64)	13.35 (7.86–23.43)	1709 (1005–2999)	148.4 (77.9–309.5)
β-Cryptoxanthin	480 (142–984)	4.48 (1.79–7.81)	2.30 (0.79–4.40)	294 (101–563)	46.5 (16.5–159.1)
α-Carotene	283 (56–751)	5.29 (0.53–11.88)	2.50 (0.29–6.48)	320.0 (36.5–828.8)	86.4 (10.9–467.7)
Total β-Carotene	1812 (1081–3192)	39.54 (23.0–59.01)	19.13 (11.02–35.94)	2449 (1411–4600)	150.4 (65.5–264.9)
Lycopene	13146 (5143–26876)	20.02 (9.57–34.55)	10.97 (5.16–19.22)	1404 (660–2460)	11.5 (4.0–25.4)
Phytoene *	2697 (1560–4207)	11.37 (4.30–20.00)	5.67 (1.94–11.14)	726 (248–1425)	24.5 (10.7–62.4)
Phytofluene *	610 (310–953)	3.04 (0–8.08)	1.77 (0–4.60)	227 (0–590)	32.1 (0–84.4)
Provitamin A carotenoids	2913 (1640–4507)	53.91 (29.88–83.0)	25.81 (14.71–49.44)	3304 (1883–6328)	122.6 (59.8–231.0)
Non-pro A carotenoids	20384 (9104–31040)	66.37(42.33–102.5)	34.38 (21.68–58.79)	4334 (2760–7513)	32.3 (17.1–91.0)

* Normal distribution.
